# Impact of directionality and correlation on contagion

**DOI:** 10.1038/s41598-018-22508-1

**Published:** 2018-03-19

**Authors:** Xin-Jian Xu, Jia-Yan Li, Xinchu Fu, Li-Jie Zhang

**Affiliations:** 1 Department of Mathematics, Shanghai University, Shanghai, 200444 China; 2 Key Laboratory of Embedded System and Service Computing (Tongji University), Ministry of Education, Shanghai, 201804 China; 3 Department of Physics, Shanghai University, Shanghai, 200444 China

## Abstract

The threshold model has been widely adopted for modelling contagion processes on social networks, where individuals are assumed to be in one of two states: inactive or active. This paper studies the model on directed networks where nodal inand out-degrees may be correlated. To understand how directionality and correlation affect the breakdown of the system, a theoretical framework based on generating function technology is developed. First, the effects of degree and threshold heterogeneities are identified. It is found that both heterogeneities always decrease systematic robustness. Then, the impact of the correlation between nodal in- and out-degrees is investigated. It turns out that the positive correlation increases the systematic robustness in a wide range of the average in-degree, while the negative correlation has an opposite effect. Finally, a comparison between undirected and directed networks shows that the presence of directionality and correlation always make the system more vulnerable.

## Introduction

Contagion processes arise broadly in biological, social, and information systems. Examples include the spread of infectious diseases^1^, the diffusion of cultural fads^2^, the outbreak of political unrest^3^ and the dissemination of rumor^4^. All these processes can be studied by contagion models, in which inactive (or susceptible) individuals are activated (or infected) by contacts with active neighbours. In general, the propagation of individual states is often characterized as either a simple contagion or a complex contagion^5^. A simple contagion is any process where the infection probability is assumed to be independent and identical across successive contacts, which is widely adopted in mathematical models of infectious diseases^6,7^. On the other hand, a complex contagion is a process where the infection probability is related to a certain critical number of exposures to infection an individual has, which usually exhibits cascade phenomena observed in social and economical systems^5,8^. Here, we are interested in complex contagion. One of the prototypes for studying such dynamics is the threshold model, which originated from the seminal work of Schelling^9^ on residential segregation, and subsequently was developed by Granovetter^10^ in the study on social influences. According to the general definition of the threshold model, an individual adopts a new product or idea only if a critical fraction^11^ or number^12^ of her friends have already been activated. This required fraction/number of adopters in the neighbourhood is defined as *threshold*.

The threshold model has been studied on undirected networks profoundly^11–21^. Although the contagion rule is simple, it turns out that the model can exhibit complex behaviour when individual difference and interaction structure are considered. Watts^11^ first studied the model with one random initiator on complex networks to examine the effects of these factors on the cascade dynamics: it was shown that heterogeneous nodal degrees enhance systemic stability compared to that of homogeneous nodal degrees. Threshold heterogeneity, however, has a contrary effect. Gleeson and Cahalane^14^ extended Watts’ model to a finite number of initiators. They found that the varying seed size has a broad impact on the cascade transition as a function of the average degree *z* of nodes, even making the transition to be discontinuous for relatively small values of *z*. Singh *et al.*^18^ also demonstrated the effect of seed selection on the cascade condition and final prevalence, for instance, selecting seeds by their degrees (highest first) results in the largest (as well as fastest) spread in Erdös-Rényi (ER)^22^ networks.

However, most contagion processes are directed such as communication in email networks^23^, diffusion in financial networks^24^, information sharing in Twitter^25^ and opinion following in Microblog^26^. In directed networks, a node is connected to others via incoming and outgoing links. Each node receives information via incoming links and sends it via outgoing ones. The presence of directionality opens the door to features that are essentially different from those in undirected networks. Dodds and collaborators^27,28^ studied global spreading based on the propagation counts of edge-node pairs rather than just nodes. They constructed the gain ratio matrix for contagion in generalized random networks with both directed and undirected edges and degree-degree correlations, and obtained analytic expressions for the probability and expected size of global spreading events starting from a single seed or finite seeds. However, the calculation of the largest eigenvalue of the gain ratio matrix needs exact information of the combinations of in- and out-degrees of all the nodes. For complex directed networks, it is much difficult in obtaining the largest eigenvalue due to high dimension.

In this paper, we develop a theoretical framework based on generating function technology to calculate the condition and prevalence of global cascades. We study analytically and numerically the threshold model on directed Poisson and power-law networks. Similar to undirected networks^11,14^, a global cascade is not triggered in directed networks when the average in-degree *z*_in_ of nodes is either too small or too large, however, large cascades are realized within an intermediate range of *z*_in_, which is referred to as the cascade window. In contrast to undirected networks, both degree and threshold heterogeneities make directed networks more vulnerable. Moreover, if the correlations between nodal in- and out-degrees are considered, the system shows distinct behaviours in most regimes of *z*_in_: the positive correlation makes the system robust to contagion, while the negative correlation makes the system prone to failure.

## Results

In the threshold model, each node *i* can only exist in one of two discrete states: inactive or active. The rationality of *i* can be represented by a random threshold *r*_*i*_ ∈ (0, 1), which is a random variable drawn from the distribution *f* (*r*) with ${\int }_{0}^{1}\,f(r){\rm{d}}r=1$. Initially, one node is chosen randomly from the network to be active, and the others are inactive. In a directed network, a node can be influenced by its neighbours via incoming links (influenced neighbours) and influences others via outgoing links (influencing neighbours). At each time step, an inactive node *i* will be activated if the active number of its influenced neighbours *m*_*i*_ satisfies

1 $$\frac{{m}_{i}}{{k}_{i}^{{\rm{in}}}}\ge {r}_{i},$$

where ${k}_{i}^{{\rm{in}}}$ is the in-degree of *i*. Once the node is activated, it remains unchanged. If node *i* is an initial seed, it will first activate its influencing neighbours *j* whose thresholds satisfy

2 $$\frac{1}{{k}_{j}^{{\rm{in}}}}\ge {r}_{j}.$$

Due to their unstable characteristic in the one-step sense, we call these influencing neighbours vulnerable nodes^11^. In any sufficiently large network with a small number of seeds, the only way in which the seed can grow is that at least one of its influencing neighbours is vulnerable. If the network is undirected, the necessary condition for a global cascade is the existence of a connected cluster of vulnerable nodes occupying a finite fraction of the network; that is, there must exist a giant component of vulnerable nodes (GCVN). Whereas for the the directed network, the giant in-component (GINC), the giant strongly connected component (GSCC), and the giant out-component (GOUC) of vulnerable nodes appear or disappear simultaneously, any of which can be used to determine whether global cascades commence. Based on generating functions for directed networks with and without correlations between in- and out-degrees, we obtain analytic expressions for the possibility and expected size of the large cascade, as manifested in the method section.

**Figure 1:**
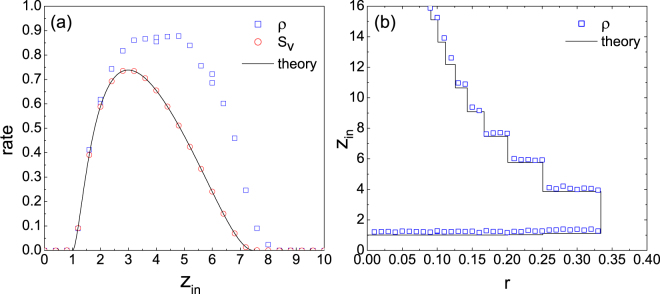
Comparison between the size of vulnerable component *S*_*v*_ and active fraction *ρ* in directed ER networks without correlation. (**a**) Values of *S*_*v*_ from Eq. (14) and simulation results of *ρ* as a function of average in-degree *z*_in_ for *r* = 0.18. (**b**) Cascade windows in the (*r*, *z*_in_) plane inside which the breakdown of the system is observed. In simulation, a global cascade is justified if a large value of *ρ* results from a small *ρ*_0_ with high possibility.

Let us start from the simplest case that all the nodes have identical threshold and nodal in- and out-degrees follow Poisson distributions without correlation. According to the model definition, whether a node to be active or not depends heavily on its in-degree. For the whole network, we shall focus on the dependence of the GSCC of vulnerable nodes on the average in-degree *z*_in_. Figure 1(a) shows the size *S*_*v*_ of the GSCC of vulnerable nodes and the fraction *ρ* of active nodes as a function of *z*_in_ in directed ER networks. Although *ρ* is larger than *S*_*v*_ in a wide range of *z*_in_, they occur and fade out simultaneously; that is, the cascade transition can happen either in the lower- or higher-connectivity regime. Nevertheless, the results of the transitions are distinct: in the lower-connectivity regime, the cascade propagation is limited by network sparsity. Any increase of *z*_in_ will enhance the possibility of propagation, and finally causes the lower transition to occur which makes the system shift from a stable state to a vulnerable one; in the higher-connectivity regime, on the contrary, a node is surrounded by many inactive neighbors due to high network density, any increase of *z*_in_ gives rise to its local stability, and finally leads to the higher transition which makes the system shift from a vulnerable state to a stable one. Thus, only within an intermediate range of *z*_in_ can a global cascade be triggered given a proper value of the threshold. As demonstrated in Fig. 1(b), the cascade condition (Eq. (13)) is expressed as a boundary in the (*r*, *z*_in_) plane (solid line). For comparison, simulation results of *ρ* (open squares) outline the window inside which large cascades occur, which are averaged over 100 realizations of the systems with the same parameter settings. Although the size of simulating networks is finite (*N* = 10000), analytical and actual boundaries agree well.

### The impact of heterogeneity

**Figure 2:**
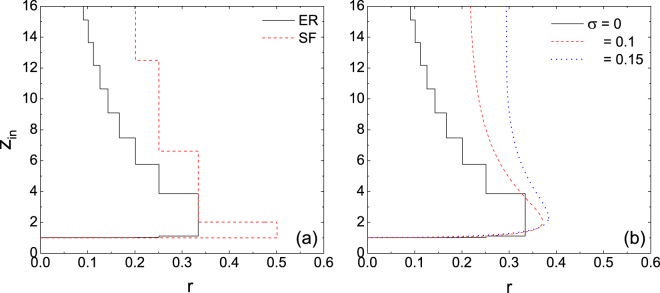
Impacts of degree and threshold heterogeneities on the cascade windows. (**a**) The dashed line represents the cascade window in directed SF networks without correlation. All the nodes have identical threshold. (**b**) The dashed and dot lines represent the cascade windows in directed ER networks without correlation, but where nodal thresholds are normally distributed with mean *r* and different SD *σ*.

Previous studies have identified the effects of degree and threshold heterogeneities^11,29^ on systematic stability by varying the distributions of nodal degrees and thresholds, for instance, an undirected network with the heterogeneous degree distribution tends to be more robust to random attacks than an undirected homogeneous network. In the present paper, the degree heterogeneity is realized by the power-law distributions of the in-degree *k*^in^ and out-degree *k*^out^, hence scale free (SF)^30^. Whereas for the threshold heterogeneity, we adopt the normal distribution with mean *r* and standard deviation *σ*. Figure 2(a) presents the cascade window in directed SF networks and compare it to directed ER networks. In both networks, nodal thresholds are identical. In contrast to the undirected situation, the directed SF network is more vulnerable than the directed ER network to random attacks. It results from the heavy dependence of the cascade condition on the average in-degree *z*_in_. Different from the directed ER network which is sharply peaked around a well defined *z*_in_, the directed SF network is highly right-skewed; that is, the number of small in-degree nodes in the directed SF network is larger than that in the directed ER network, which yields more vulnerable nodes in the directed SF network according to Eq. (2), and therefore gives rise to cascading. Figure 2(b) shows the comparison of the cascade windows for identical (solid line) and normally distributed thresholds (dashed and dot lines). Meanwhile, the distributions of *k*^in^ and *k*^out^ are Poisson. As *σ* increases, the normal distribution becomes wide, and the fraction of nodes whose thresholds may be far from the mean. The nodes with thresholds below average will be easily activated while those with thresholds above average are difficult to be activated. When the seed fraction is very small, the nodes with thresholds below average plays an overwhelming role in contagion compared to those with thresholds above average^20^. Thus, the heterogeneity of nodal thresholds increases the likelihood of large cascades.

### The impact of correlation

**Figure 3:**
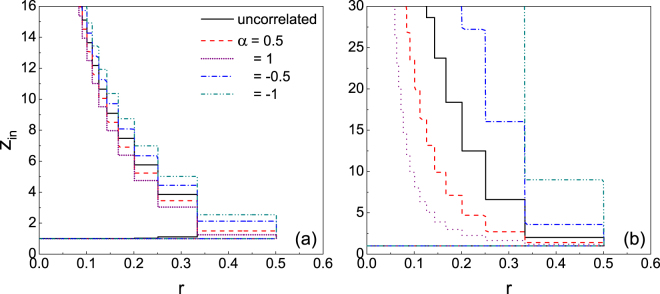
Impacts of in- and out-degree correlations on the cascade windows in directed ER (**a**) and SF (**b**) networks. The colored lines enclose the regions of the (*r*, *z*_in_) plane in which the cascade condition (Eq. (15)) is satisfied.

In directed networks, the correlation between in- and out-degrees is an important characteristic and has been the focus of many studies including robustness^31^, controllability^32^ and synchronization^33^. In the present paper, the correlation between in-degree ${k}_{i}^{{\rm{in}}}$ and out-degree ${k}_{i}^{{\rm{out}}}$ of node *i* is assumed to take the form ${k}_{i}^{{\rm{out}}}\sim {({k}_{i}^{{\rm{in}}})}^{\alpha }$, where *α* is a tunable constant^34^. *α* > 0 corresponds to the positive correlation between ${k}_{i}^{{\rm{out}}}$ and ${k}_{i}^{{\rm{in}}}$, i.e., a node of high in-degree has high out-degree as well; *α* < 0 refers to the negative correlation between ${k}_{i}^{{\rm{out}}}$ and ${k}_{i}^{{\rm{in}}}$, i.e., a node of high in-degree has small out-degree instead. Intuitively, the negative correlation between *k*^out^ and *k*^in^ could weaken the robustness of the system, since the possibility for a node of small *k*^in^ being vulnerable is high, meanwhile the large *k*^out^ makes it having many influencing neighbours. Hence, it facilitates cascade propagation. Whereas for the positive correlation, even though a node of small *k*^in^ may be vulnerable, the assortative small *k*^out^ limits the number of influencing neighbours. It therefore has difficulty in propagating any influence and the systematic robustness is enhanced. Figure 3 demonstrates the effect of *α* on the cascade windows in directed ER and SF networks over a wide range of both *r* and *z*_in_. Compared to the directed ER network, the directed SF network is largely affected by the correlation between in- and out-degrees. In particular, the larger the value of *α* is, the more robustness the system becomes, either for *α* > 0 or *α* < 0. The only exception is the interval *z*_in_ ∈ (1.1, 1.5) where the positive correlation could decrease the robustness of the directed ER network. When *z*_in_ is very small, the network is poorly connected and the cascade propagation is limited. Therefore, nodes of large degree are responsible for triggering large cascades. Compared to the uncorrelated ER network, the positive correlations between in- and out-degrees of these nodes increase the likelihood of propagation, hence the decrease of the robustness of the system.

### Comparison with undirected networks

**Figure 4:**
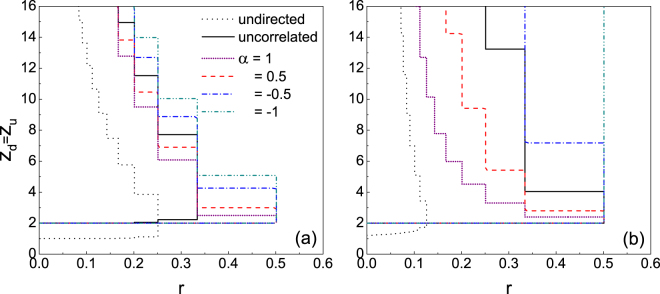
Comparison of the cascade windows in directed networks with those in undirected networks for *z*_d_ = *z*_u_. Nodal degree distribution of the undirected network and in- and out-degrees distributions of the directed network are simultaneously Poisson (**a**) and power-law (**b**), respectively.

**Figure 5:**
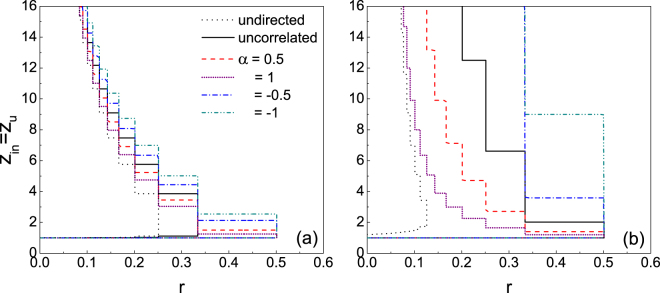
Comparison of the cascade windows in directed networks with those in undirected networks for *z*_in_ = *z*_u_. Nodal degree distribution of the undirected network and in- and out-degrees distributions of the directed network are simultaneously Poisson (**a**) and power-law (**b**), respectively.

When comparing the robustness of directed networks with undirected networks, we consider two situations. One is that the average degree *z*_d_ (=*z*_in_ + *z*_out_) of the directed network equals the average degree *z*_u_ of the undirected network, i.e., the total number of links of the directed network is same to that of the undirected network. The other is the equivalence of *z*_in_ and *z*_u_, i.e., the total number of links of the directed network is twice of that of the undirected network. Figure 4 shows the comparison of cascade windows in directed and undirected networks for *z*_d_ = *z*_u_. The lowest boundaries of large cascades for both directed ER and SF networks are *z*_d_ = 2 (consistent with *z*_in_ = 1). So long as *z*_d_ > 2, the size of the window in directed networks is larger than that in undirected networks; that is, a directed network is more vulnerable than a undirected one with respect to network connectivity. Given a proper value of the threshold *r*, whether a node in the undirected network is vulnerable depends on its degree *z*_u_, whereas for the directed network the nodal vulnerableness is dependent on its in-degree *z*_in_. In the case of *z*_d_ = *z*_u_, one has *z*_in_ = *z*_u_/2. According to Eq. (2), the directed network has a larger number of vulnerable nodes than the undirected one, hence the less stability of the system. Figure 5 shows the comparison of the cascade windows in directed and undirected networks for *z*_in_ = *z*_u_. Again, one notices similar behaviour regardless of the nodal in- and out-degree distributions and correlations. In the case of *z*_in_ = *z*_u_, the possibility for a node being vulnerable in the directed network is the same as that in the undirected network. Meanwhile, the extra amount of outgoing links (*z*_out_ = *z*_u_) of the directed network enables it to influence more neighbours compared to the undirected network, hence the promotion of propagation.

## Discussion

The investigation of structure and dynamics of social networks has attracted increasing attention from applied mathematicians, statistical physicists, and computer scientists over the past decades^35^. Of high interest is a broad range of contagion processes taking place over underline networks. In spite of its simplicity, the threshold model has attracted much attention with practical applications in viral marketing^36^, emotion transitivity^37^ and risk perception^38^. However, very few studies have considered asymmetry of social interactions. In this paper, we extended the threshold model to directed ER and SF networks in which each node is connected to others via incoming and outgoing links with and without correlations.

Based on generating function technology, we have developed a theoretical framework for analyzing the threshold model on large directed networks. Through the calculation of the size of GSCC of vulnerable nodes, we obtained the condition and prevalence of large cascades in the directed network, which differ from those in the undirected network. For instance, both heterogeneities of nodal degrees and thresholds could decrease the systematic robustness. Moreover, the correlation between nodal in- and out-degrees has mixed effects on systemic stability: when directed networks are heterogeneous, the positive correlation increases the robustness, while the negative correlation decreases the robustness; when the directed networks are homogeneous, the above results hold when network connectivity is relatively high, nevertheless, the positive correlation decreases the systematic robustness when network connectivity is very low. Finally, by comparing the robustness of the threshold model on directed and undirected networks, it turns out that the presence of directionality always makes the system more vulnerable, regardless of the distributions of in- and out-degrees as well as correlations between them. These results complement previous studies^27,28^.

We note, however, social dynamics is more complex^39^. To study contagion in realistic networks, one needs to generalize the present framework by incorporating more physical and structural properties. The comprehensive investigation of the frequency and size of large cascades through theoretical and empirical approaches is of significant interest.

## Methods

Given a directed network, the joint probability distribution of a node of in-degree *k*^in^ and out-degree *k*^out^ is defined by *p*(*k*^in^, *k*^out^). According to Eq. (2), a node of in-degree *k*^in^ is vulnerable with probability $\rho ({k}^{{\rm{in}}})=P(r\le \tfrac{1}{{k}^{{\rm{in}}}})$. Therefore, the generating function for the joint degree distribution of vulnerable nodes is ${g}_{00}(x,y)=$ ${\sum }_{{k}^{{\rm{in}}},{k}^{{\rm{out}}}}\,\rho ({k}^{{\rm{in}}})p({k}^{{\rm{in}}},{k}^{{\rm{out}}}){x}^{{k}^{{\rm{in}}}}{y}^{{k}^{{\rm{out}}}}$, based on which one has two generating functions for in- and out-degree distributions of vulnerable nodes,

3 $${g}_{00}(x,\mathrm{1)}=\sum _{{k}^{{\rm{in}}},{k}^{{\rm{out}}}}\,\rho ({k}^{{\rm{in}}})p({k}^{{\rm{in}}},{k}^{{\rm{out}}}){x}^{{k}^{{\rm{in}}}}\,{\rm{and}}\,{g}_{00}\mathrm{(1,}y)=\sum _{{k}^{{\rm{in}}},{k}^{{\rm{out}}}}\,\rho ({k}^{{\rm{in}}})p({k}^{{\rm{in}}},{k}^{{\rm{out}}}){y}^{{k}^{{\rm{out}}}},$$

respectively. To describe propagation from one node to another, one also requires generating functions for the joint excess degree of vulnerable nodes either approaching a random node or originated from the node,

4 $${g}_{01}(x,y)=\sum _{{k}^{{\rm{in}}},{k}^{{\rm{out}}}}\,\frac{\rho ({k}^{{\rm{in}}}){k}^{{\rm{out}}}p({k}^{{\rm{in}}},{k}^{{\rm{out}}})}{{z}_{{\rm{out}}}}{x}^{{k}^{{\rm{in}}}}{y}^{{k}^{{\rm{out}}}-1}=\frac{1}{{z}_{{\rm{out}}}}\frac{\partial {g}_{00}(x,y)}{\partial y}$$

and

5 $${g}_{10}(x,y)=\sum _{{k}^{{\rm{in}}},{k}^{{\rm{out}}}}\,\frac{\rho ({k}^{{\rm{in}}}){k}^{{\rm{in}}}p({k}^{{\rm{in}}},{k}^{{\rm{out}}})}{{z}_{{\rm{in}}}}{x}^{{k}^{{\rm{in}}}-1}{y}^{{k}^{{\rm{out}}}}=\frac{1}{{z}_{{\rm{in}}}}\frac{\partial {g}_{00}(x,y)}{\partial x},$$

respectively, where ${z}_{{\rm{in}}}={\sum }_{{k}^{{\rm{in}}},{k}^{{\rm{out}}}}\,{k}^{{\rm{in}}}p({k}^{{\rm{in}}},{k}^{{\rm{out}}})$ is the average in-degree of nodes and ${z}_{{\rm{out}}}={\sum }_{{k}^{{\rm{in}}},{k}^{{\rm{out}}}}\,{k}^{{\rm{out}}}p({k}^{{\rm{in}}},{k}^{{\rm{out}}})$ is the average out-degree, hence *z*_in_ = *z*_out_ = *z*_d_/2. Based on *g*_01_(*x*, *y*) and *g*_10_(*x*, *y*), one has generating functions for the excess in- and out-degree distributions of vulnerable nodes,

6 $${g}_{01}(x,\mathrm{1)}=\sum _{{k}^{{\rm{in}}},{k}^{{\rm{out}}}}\,\frac{\rho ({k}^{{\rm{in}}}){k}^{{\rm{out}}}p({k}^{{\rm{in}}},{k}^{{\rm{out}}})}{{z}_{{\rm{out}}}}{x}^{{k}^{{\rm{in}}}}\,{\rm{and}}\,{g}_{10}\mathrm{(1},y)=\sum _{{k}^{{\rm{in}}},{k}^{{\rm{out}}}}\,\frac{\rho ({k}^{{\rm{in}}}){k}^{{\rm{in}}}p({k}^{{\rm{in}}},{k}^{{\rm{out}}})}{{z}_{{\rm{in}}}}{y}^{{k}^{{\rm{out}}}},$$

respectively. To analyze the properties of vulnerable clusters, we introduce analogous generating functions for size distributions of in- and out-components of vulnerable nodes,

7 $${\varphi }_{0}(x)=1-{g}_{00}\mathrm{(1},\mathrm{1)}+x\,\sum _{{k}^{{\rm{in}}},{k}^{{\rm{out}}}}\,\rho ({k}^{{\rm{in}}})p({k}^{{\rm{in}}},{k}^{{\rm{out}}})\,{[{\varphi }_{1}(x)]}^{{k}^{{\rm{in}}}}=1-{g}_{00}\mathrm{(1},\mathrm{1)}+x{g}_{00}({\varphi }_{1}(x),\mathrm{1)}$$

and

8 $${\phi }_{0}(y)=1-{g}_{00}\mathrm{(1},\mathrm{1)}+y\,\sum _{{k}^{{\rm{in}}},{k}^{{\rm{out}}}}\,\rho ({k}^{{\rm{in}}})p({k}^{{\rm{in}}},{k}^{{\rm{out}}})\,{[{\phi }_{1}(y)]}^{{k}^{{\rm{out}}}}=1-{g}_{00}\mathrm{(1},\mathrm{1)}+y{g}_{00}\mathrm{(1},{\phi }_{1}(y)),$$

respectively. *ϕ*_1_(*x*) and *φ*_1_(*y*) are corresponding generating functions for the sizes of the in-component of vulnerable nodes arriving at a random node and the out-component leaving from the node, defined by

9 $${\varphi }_{1}(x)=1-{g}_{01}\mathrm{(1},\mathrm{1)}+x\,\sum _{{k}^{{\rm{in}}},{k}^{{\rm{out}}}}\,\frac{\rho ({k}^{{\rm{in}}}){k}^{{\rm{out}}}p({k}^{{\rm{in}}},{k}^{{\rm{out}}})}{{z}_{{\rm{out}}}}{[{\varphi }_{1}(x)]}^{{k}^{{\rm{in}}}}=1-{g}_{01}\mathrm{(1},\mathrm{1)}+x{g}_{01}({\varphi }_{1}(x),\mathrm{1)}$$

and

10 $${\phi }_{1}(y)=1-{g}_{10}\mathrm{(1},\mathrm{1)}+y\,\sum _{{k}^{{\rm{in}}},{k}^{{\rm{out}}}}\,\frac{\rho ({k}^{{\rm{in}}}){k}^{{\rm{in}}}p({k}^{{\rm{in}}},{k}^{{\rm{out}}})}{{z}_{{\rm{in}}}}{[{\phi }_{1}(y)]}^{{k}^{{\rm{out}}}}=1-{g}_{10}\mathrm{(1},\mathrm{1)}+y{g}_{10}\mathrm{(1},{\phi }_{1}(y\mathrm{))}.$$

respectively.

### Condition for global cascades without correlation

In the directed network, the GINC, GSCC and GOUC of vulnerable nodes appear or disappear simultaneously^31^. Being interested in propagation along directed links, we shall investigate the GOUC of vulnerable nodes. From Eq. (8), it follows that ${\phi ^{\prime} }_{0}\mathrm{(1)}=$ ${g}_{00}\mathrm{(1},{\phi }_{1}\mathrm{(1))}+\frac{\partial {g}_{00}\mathrm{(1},{\phi }_{1}\mathrm{(1))}}{\partial y}{\phi ^{\prime} }_{1}\mathrm{(1)}$, which is the average size of the GOUC of vulnerable nodes. Noting that *φ*_1_(1) = 1, one obtains

11 $${\phi ^{\prime} }_{0}\mathrm{(1)}={g}_{00}\mathrm{(1},\mathrm{1)}+\frac{\partial {g}_{00}\mathrm{(1},\mathrm{1)}}{\partial y}{\phi ^{\prime} }_{1}\mathrm{(1}).$$

Similarly, one has ${\phi ^{\prime} }_{1}\mathrm{(1)}={g}_{10}\mathrm{(1},\mathrm{1)}+\frac{\partial {g}_{10}\mathrm{(1},\mathrm{1)}}{\partial y}{\phi ^{\prime} }_{1}\mathrm{(1)}$, which yields ${\phi ^{\prime} }_{1}\mathrm{(1)}={g}_{10}\mathrm{(1},\mathrm{1)}/[1-\frac{\partial {g}_{10}\mathrm{(1},\mathrm{1)}}{\partial y}]$. Thus, Eq. (11) can be rewritten as

12 $${\phi ^{\prime} }_{0}\mathrm{(1)}={g}_{00}\mathrm{(1},\mathrm{1)}+\frac{\frac{\partial {g}_{00}\mathrm{(1},\mathrm{1)}}{\partial y}{g}_{10}\mathrm{(1},\mathrm{1)}}{1-\frac{\partial {g}_{10}\mathrm{(1},\mathrm{1)}}{\partial y}},$$

which diverges as $\frac{\partial {g}_{10}\mathrm{(1},\mathrm{1)}}{\partial y}=1$, i.e,

13 $$\sum _{{k}^{{\rm{in}}},{k}^{{\rm{out}}}}\,{k}^{{\rm{in}}}{k}^{{\rm{out}}}\rho ({k}^{{\rm{in}}})p({k}^{{\rm{in}}},{k}^{{\rm{out}}})={z}_{{\rm{in}}}.$$

In analogy to undirected networks^11^, the above equation determines whether global cascades commence. To calculate the size of the GSCC of vulnerable nodes, we randomly choose a node of in-degree *k*^in^ and out-degree *k*^out^. The probability that there is at least one path from the GSCC of vulnerable nodes to the node via any incoming link is $1-{[{\varphi }_{1}\mathrm{(1)]}}^{{k}^{{\rm{in}}}}$. Meanwhile, the probability that there is at least one path from the node to the GSCC of vulnerable nodes via any outgoing link is $1-{[{\phi }_{1}\mathrm{(1)]}}^{{k}^{{\rm{out}}}}$. Therefore, the size of the GSCC of vulnerable nodes is

14 $$\begin{array}{rcl}{S}_{v} & = & \sum _{{k}^{{\rm{in}}},{k}^{{\rm{out}}}}\,\rho ({k}^{{\rm{in}}})p({k}^{{\rm{in}}},{k}^{{\rm{out}}}\mathrm{)\{1}-{[{\varphi }_{1}\mathrm{(1)]}}^{{k}^{{\rm{in}}}}\mathrm{\}\{1}-{[{\phi }_{1}\mathrm{(1)]}}^{{k}^{{\rm{out}}}}\}\\  & = & \sum _{{k}^{{\rm{in}}},{k}^{{\rm{out}}}}\,\rho ({k}^{{\rm{in}}})p({k}^{{\rm{in}}},{k}^{{\rm{out}}})-\sum _{{k}^{{\rm{in}}},{k}^{{\rm{out}}}}\,\rho ({k}^{{\rm{in}}})p({k}^{{\rm{in}}},{k}^{{\rm{out}}})[{\varphi }_{1}{\mathrm{(1)]}}^{{k}^{{\rm{in}}}}\\  &  & -\sum _{{k}^{{\rm{in}}},{k}^{{\rm{out}}}}\,\rho ({k}^{{\rm{in}}})p({k}^{{\rm{in}}},{k}^{{\rm{out}}})[{\phi }_{1}{\mathrm{(1)]}}^{{k}^{{\rm{out}}}}\\  &  & +\sum _{{k}^{{\rm{in}}},{k}^{{\rm{out}}}}\,\rho ({k}^{{\rm{in}}})p({k}^{{\rm{in}}},{k}^{{\rm{out}}})[{\varphi }_{1}{\mathrm{(1)]}}^{{k}^{{\rm{in}}}}{[{\phi }_{1}\mathrm{(1)]}}^{{k}^{{\rm{out}}}}.\end{array}$$

### Condition for global cascades with correlation

In the case that the in-degree *k*^in^ and out-degree *k*^out^ of a node are correlated, we adopt the form *k*^out^ = *c*(*k*^in^)^*α*^ ^34^. According to the normalization one obtains $c={z}_{{\rm{in}}}/[{\sum }_{{k}^{{\rm{in}}}}\,{({k}^{{\rm{in}}})}^{\alpha }p({k}^{{\rm{in}}})]$ with $p({k}^{{\rm{in}}})={\sum }_{{k}^{{\rm{out}}}}\,p({k}^{{\rm{in}}},{k}^{{\rm{out}}})$. Thereby, the cascade condition can be rewritten as

15 $$\sum _{{k}^{{\rm{in}}}}\,c{({k}^{{\rm{in}}})}^{\alpha +1}\rho ({k}^{{\rm{in}}})p({k}^{{\rm{in}}})={z}_{{\rm{in}}},$$

and the corresponding size of the GSCC of vulnerable nodes is

16 $$\begin{array}{rcl}{S}_{v} & = & \sum _{{k}^{{\rm{in}}}}\,\rho ({k}^{{\rm{in}}})p({k}^{{\rm{in}}})\{1-{[{\varphi }_{1}\mathrm{(1)]}}^{{k}^{{\rm{in}}}}\}\{1-{[{\phi }_{1}\mathrm{(1)]}}^{c{({k}^{{\rm{in}}})}^{\alpha }}\}\\  & = & \sum _{{k}^{{\rm{in}}}}\,\rho ({k}^{{\rm{in}}})p({k}^{{\rm{in}}})-\sum _{{k}^{{\rm{in}}}}\,\rho ({k}^{{\rm{in}}})p({k}^{{\rm{in}}})[{\varphi }_{1}{\mathrm{(1)]}}^{{k}^{{\rm{in}}}}\\  &  & -\sum _{{k}^{{\rm{in}}}}\,\rho ({k}^{{\rm{in}}})p({k}^{{\rm{in}}})[{\phi }_{1}{\mathrm{(1)]}}^{c{({k}^{{\rm{in}}})}^{\alpha }}\\  &  & +\sum _{{k}^{{\rm{in}}}}\,\rho ({k}^{{\rm{in}}})p({k}^{{\rm{in}}})[{\varphi }_{1}{\mathrm{(1)]}}^{{k}^{{\rm{in}}}}{[{\phi }_{1}\mathrm{(1)]}}^{c{({k}^{{\rm{in}}})}^{\alpha }},\end{array}$$

with

17 $${\varphi }_{1}\mathrm{(1)}=1-{g}_{01}\mathrm{(1},\mathrm{1)}+\sum _{{k}^{{\rm{in}}}}\,\frac{c\rho ({k}^{{\rm{in}}})\,{({k}^{{\rm{in}}})}^{\alpha }p({k}^{{\rm{in}}})}{{z}_{{\rm{out}}}}{[{\varphi }_{1}\mathrm{(1)]}}^{{k}^{{\rm{in}}}}$$

and

18 $${\phi }_{1}\mathrm{(1)}=1-{g}_{10}\mathrm{(1},\mathrm{1)}+\sum _{{k}^{{\rm{in}}}}\,\frac{\rho ({k}^{{\rm{in}}}){k}^{{\rm{in}}}p({k}^{{\rm{in}}})}{{z}_{{\rm{in}}}}{[{\phi }_{1}(y)]}^{c{({k}^{{\rm{in}}})}^{\alpha }}.$$

## References

[1] Anderson RM, May RM (1991). Infectious Diseases of Humans: Dynamics and Control.

[2] Bikhchandani S, Hirshleifer D, Welch I (1992). A theory of fads, fashion, custom, and cultural change as informational cascades. J. Polit. Econ..

[3] Lohmann S (1994). The dynamics of informational cascades. World Polit..

[4] Pittel B (1987). On spreading a rumor. SIAM J. Appl. Math..

[5] Porter, M. A. & Gleeson, J. P. Dynamical systems on networks: a tutorial (Springer, 2016).

[6] Keeling MJ, Rohani P (2007). Modeling infectious diseases in humans and animals.

[7] Wang Z (2016). Statistical physics of vaccination. Phys. Rep..

[8] Zhang Z-K (2016). Dynamics of information diffusion and its applications on complex networks. Phys. Rep..

[9] Schelling TC (1971). Dynamic models of segregation. J. Math. Soc..

[10] Granovetter M (1978). Threshold models of collective behavior. Am. J. Sociol..

[11] Watts DJ (2002). A simple model of global cascades on random networks. Proc. Natl. Acad. Sci. USA.

[12] Centola D, Macy M (2007). Complex contagions and the weakness of long ties. Am. J. Sociol..

[13] Dodds PS, Watts DJ (2004). Universal Behavior in a Generalized Model of Contagion. Phys. Rev. Lett..

[14] Gleeson JP, Cahalane DJ (2007). Seed size strongly affects cascades on random networks. Phys. Rev. E.

[15] Centola D, Eguíluz VM, Macy MW (2007). Cascade dynamics of complex propagation. Physica A.

[16] Hackett A, Melnik S, Gleeson JP (2011). Cascades on a class of clustered random networks. Phys. Rev. E.

[17] Dodds PD, Harris KD, Danforth CM (2013). Limited Imitation Contagion on Random Networks. Phys. Rev. Lett..

[18] Singh P, Sreenivasan S, Szymansko BK, Korniss G (2013). Threshold-limited spreading in social networks with multiple initiators. Sci. Rep..

[19] Lim S, Jung I, Lee S, Jung K (2015). Analysis of information diffusion for threshold models on arbitrary networks. Eur. Phy. J. B.

[20] Karampourniotis PD, Sreenivasan S, Szymanski BK, Korniss G (2015). The Impact of heterogeneous thresholds on social contagion with multiple initiators. PLoS One.

[21] Guo Q, Jiang X, Lei Y, Li M, Ma Y, Zheng Z (2015). Two-stage effects of awareness cascade on epidemic spreading in multiplex networks. Phys. Rev. E.

[22] Erdös P, Rényi A (1959). On random graphs. Publ. Math. Debrecen.

[23] Newman MEJ, Forrest S, Balthrop J (2002). Email networks and the spread of computer viruses. Phys. Rev. E.

[24] Gai P, Kapadia S (2010). Contagion in financial networks. Proc. Roy. Soc. A.

[25] Kwak, H., Lee, C., Park, H. & Moon S. What is Twitter, a social network or a news media? In Proceedings of the 19th International Conference on World Wide Web, pp. 591–600 (ACM North Carolina, 2010).

[26] Chen, Z., Liu, P., Wang, X. & Gu, Y. Follow whom? Chinese users have different choice. arXiv:1212.0167.

[27] Dodds PS, Harris KD, Payne JL (2011). Direct, physically motivated derivation of the contagion condition for spreading processes on generalized random networks. Phys. Rev. E.

[28] Payne JL, Harris KD, Dodds PS (2011). Exact solutions for social and biological contagion models on mixed directed and undirected, degree-correlated random networks. Phys. Rev. E.

[29] Huang W-M, Zhang L-J, Xu X-J, Fu X (2017). Contagion on complex networks with persuasion. Sci. Rep..

[30] Catanzaro M, Boguñá M, Pastor-Satorras R (2005). Generation of uncorrelated random scale-free networks. Phys. Rev. E.

[31] Liu X, Stanley HE, Gao J (2016). Breakdown of interdependent directed networks. Proc. Natl. Acad. Sci. USA.

[32] Liu X, Pan L, Stanley HE, Gao J (2017). Controllability of giant connected components in a directed network. Phys. Rev. E.

[33] Skardal PS, Taylor D, Sun J (2016). Optimal synchronization of directed complex networks. Chaos.

[34] Mislove, A., Marcon, M., Gummadi, K. P., Druschel, P. & Bhattacharjee, B. Measurement and analysis of online social networks. in Proceedings of the 7th ACM SIGCOMM Conference on Internet Measurement, pp. 29–42 (ACM New York, 2007).

[35] Lazer D (2009). Computational social science. Science.

[36] Leskovec J, Adamic LA, Huberman BA (2007). The Dynamics of Viral Marketing. *ACM Trans*. Web.

[37] Kramer ADI, Guillory JE, Hancock JT (2014). Experimental evidence of massive-scale emotional contagion through social networks. Proc. Natl. Acad. Sci. USA.

[38] Gao C, Liu J (2017). Network-based modeling for characterizing human collective behaviors during extreme events. IEEE Trans. Syst. Man Cybbern.: Syst..

[39] Castellano C, Fortunato S, Loreto V (2009). Statistical physics of social dynamics. Rev. Mod. Phys..

